# Supplementary material for: P-TRAP: a Panicle Trait Phenotyping tool
Source: BMC Plant Biol. 2013 Aug 29;13:122. doi: 10.1186/1471-2229-13-122 (PMC3848748; doi:10.1186/1471-2229-13-122)
Supplement: Additional file 7 — P-TRAP source code. The Netbeans project that contains the source code of the application. [file 1471-2229-13-122-S7.zip › javadoc.zip!/javadoc/org-biolag-particleprocessor/org/biolag/particleprocessor/Particle.html]

Particle (ParticleProcessor)


JavaScript is disabled on your browser.


- Package
- Class
- Tree
- Deprecated
- Index
- Help

*org.biolag.particleprocessor 1.0*

- Prev Class
- Next Class

- Frames
- No Frames

- All Classes

- Summary:
- Nested |
- Field |
- Constr |
- Method

- Detail:
- Field |
- Constr |
- Method


org.biolag.particleprocessor

## Class Particle

- java.lang.Object
- - org.biolag.particleprocessor.Particle

- ---

    

  ```
  public class Particle
  extends java.lang.Object
  ```

  A general class for managing particles.

  Author:
  :   faroq

- - ### Nested Class Summary

    Nested Classes

    | Modifier and Type | Class and Description |
    | --- | --- |
    | `static class` | `Particle.CONTOUR_TYPE` |
  - ### Constructor Summary

    Constructors

    Constructor and Description

    `Particle()`

    `Particle(java.util.ArrayList<java.awt.Point> points)`

    `Particle(org.w3c.dom.Element particle)`

    Create a particle from an XML `Element`.

    `Particle(Particle particle)`

    Copy constructor creates a clone particle of the input.
  - ### Method Summary

    Methods

    | Modifier and Type | Method and Description |
    | --- | --- |
    | `void` | `addConcavePoint(ConcavePoint p)` |
    | `void` | `addContourPoint(java.awt.Point p)` |
    | `double` | `area()` |
    | `void` | `createDummyParticle(int radius, java.awt.Point center)` For additional editing a dummy particle is an empty particle that the user creates. |
    | `double` | `getArea()` |
    | `java.awt.Rectangle` | `getBoundingbox()` |
    | `double` | `getCircularity()` |
    | `double` | `getCompactness()` |
    | `ConcavePoint` | `getConcavePoint(int index)` |
    | `java.util.ArrayList<ConcavePoint>` | `getConcaves()` |
    | `Particle.CONTOUR_TYPE` | `getContour_type()` |
    | `java.util.ArrayList<java.awt.Point>` | `getContour()` |
    | `java.awt.Point` | `getContourPoint(int i)` |
    | `java.util.ArrayList<java.util.ArrayList<ConcavePoint>>` | `getCurves()` |
    | `double` | `getEllipticity()` |
    | `double` | `getHeight()` |
    | `double` | `getIntensityArea()` |
    | `java.awt.Point` | `getMajorAxisAnchorPoint1()` |
    | `java.awt.Point` | `getMajorAxisAnchorPoint2()` |
    | `int` | `getMaxx()` |
    | `int` | `getMaxy()` |
    | `java.awt.Point` | `getMinorAxisAnchorPoint1()` |
    | `java.awt.Point` | `getMinorAxisAnchorPoint2()` |
    | `int` | `getMinx()` |
    | `int` | `getMiny()` |
    | `double` | `getOrientation()` |
    | `java.awt.Point` | `getPoint(int index)` |
    | `java.util.ArrayList<java.awt.Point>` | `getPoints()` |
    | `java.awt.Point` | `getPosition()` |
    | `double` | `getPrimeter()` |
    | `double` | `getProportionality()` |
    | `double` | `getWidth()` |
    | `boolean` | `isDummy()` |
    | `boolean` | `isIsfixed()` |
    | `double` | `perimeter()` An accurate estimation of the particle perimeter. |
    | `void` | `removeDuplicates()` Remove duplicates points if found. |
    | `void` | `setArea(double area)` |
    | `void` | `setBoundingbox(java.awt.Rectangle boundingbox)` |
    | `void` | `setCircularity(double circularity)` |
    | `void` | `setCompactness(double comapctness)` |
    | `void` | `setConcaves(java.util.ArrayList<ConcavePoint> concaves)` |
    | `void` | `setContour_type(Particle.CONTOUR_TYPE contour_type)` |
    | `void` | `setContour(java.util.ArrayList<java.awt.Point> contour)` |
    | `void` | `setDummy(boolean dummy)` |
    | `void` | `setEllipticity(double ellipticity)` |
    | `void` | `setHeight(double height)` |
    | `void` | `setIntensityArea(double intensityArea)` |
    | `void` | `setIsfixed(boolean isfixed)` |
    | `void` | `setMajorAxisAnchorPoint1(java.awt.Point MajorAxisAnchorPoint1)` |
    | `void` | `setMajorAxisAnchorPoint2(java.awt.Point MajorAxisAnchorPoint2)` |
    | `void` | `setMaxx(int maxx)` |
    | `void` | `setMaxy(int maxy)` |
    | `void` | `setMinorAxisAnchorPoint1(java.awt.Point MinorAxisAnchorPoint1)` |
    | `void` | `setMinorAxisAnchorPoint2(java.awt.Point MinorAxisAnchorPoint2)` |
    | `void` | `setMinx(int minx)` |
    | `void` | `setMiny(int miny)` |
    | `void` | `setOrientation(double orientation)` |
    | `void` | `setPoints(java.util.ArrayList<java.awt.Point> points)` |
    | `void` | `setPosition(java.awt.Point position)` |
    | `void` | `setPrimeter(double primeter)` |
    | `void` | `setProportionality(double proportionality)` |
    | `void` | `setWidth(double width)` |
    | `java.awt.Polygon` | `toPolygon()` Convert the particle contour to a polygon. |
    | `java.lang.String` | `toString()` In general this functions produces an XML entry of the particle. |

    - ### Methods inherited from class java.lang.Object

      `clone, equals, finalize, getClass, hashCode, notify, notifyAll, wait, wait, wait`

- - ### Constructor Detail


    - #### Particle

      ```
      public Particle()
      ```


    - #### Particle

      ```
      public Particle(java.util.ArrayList<java.awt.Point> points)
      ```


    - #### Particle

      ```
      public Particle(org.w3c.dom.Element particle)
      ```

      Create a particle from an XML `Element`.

      Parameters:
      :   `partcle` - the XML `Element` object.


    - #### Particle

      ```
      public Particle(Particle particle)
      ```

      Copy constructor creates a clone particle of the input.

      Parameters:
      :   `particle` - the input particle
  - ### Method Detail


    - #### createDummyParticle

      ```
      public void createDummyParticle(int radius,
                             java.awt.Point center)
      ```

      For additional editing a dummy particle is an empty particle that the
      user creates.

      Parameters:
      :   `radius` - the radius of the particle
      :   `center` - the center of the particle


    - #### isDummy

      ```
      public boolean isDummy()
      ```


    - #### setDummy

      ```
      public void setDummy(boolean dummy)
      ```


    - #### isIsfixed

      ```
      public boolean isIsfixed()
      ```


    - #### setIsfixed

      ```
      public void setIsfixed(boolean isfixed)
      ```


    - #### getPoints

      ```
      public java.util.ArrayList<java.awt.Point> getPoints()
      ```


    - #### setPoints

      ```
      public void setPoints(java.util.ArrayList<java.awt.Point> points)
      ```


    - #### getProportionality

      ```
      public double getProportionality()
      ```


    - #### setProportionality

      ```
      public void setProportionality(double proportionality)
      ```


    - #### getPosition

      ```
      public java.awt.Point getPosition()
      ```


    - #### setPosition

      ```
      public void setPosition(java.awt.Point position)
      ```


    - #### getPoint

      ```
      public java.awt.Point getPoint(int index)
      ```


    - #### getContour

      ```
      public java.util.ArrayList<java.awt.Point> getContour()
      ```


    - #### setContour

      ```
      public void setContour(java.util.ArrayList<java.awt.Point> contour)
      ```


    - #### getConcaves

      ```
      public java.util.ArrayList<ConcavePoint> getConcaves()
      ```


    - #### setConcaves

      ```
      public void setConcaves(java.util.ArrayList<ConcavePoint> concaves)
      ```


    - #### getContourPoint

      ```
      public java.awt.Point getContourPoint(int i)
      ```


    - #### addContourPoint

      ```
      public void addContourPoint(java.awt.Point p)
      ```


    - #### perimeter

      ```
      public double perimeter()
      ```

      An accurate estimation of the particle perimeter.

      Returns:
      :   a double value.


    - #### area

      ```
      public double area()
      ```


    - #### addConcavePoint

      ```
      public void addConcavePoint(ConcavePoint p)
      ```


    - #### getConcavePoint

      ```
      public ConcavePoint getConcavePoint(int index)
      ```


    - #### getIntensityArea

      ```
      public double getIntensityArea()
      ```


    - #### setIntensityArea

      ```
      public void setIntensityArea(double intensityArea)
      ```


    - #### getBoundingbox

      ```
      public java.awt.Rectangle getBoundingbox()
      ```


    - #### setBoundingbox

      ```
      public void setBoundingbox(java.awt.Rectangle boundingbox)
      ```


    - #### getCircularity

      ```
      public double getCircularity()
      ```


    - #### setCircularity

      ```
      public void setCircularity(double circularity)
      ```


    - #### getCompactness

      ```
      public double getCompactness()
      ```


    - #### setCompactness

      ```
      public void setCompactness(double comapctness)
      ```


    - #### getEllipticity

      ```
      public double getEllipticity()
      ```


    - #### setEllipticity

      ```
      public void setEllipticity(double ellipticity)
      ```


    - #### getMaxx

      ```
      public int getMaxx()
      ```


    - #### setMaxx

      ```
      public void setMaxx(int maxx)
      ```


    - #### getMaxy

      ```
      public int getMaxy()
      ```


    - #### setMaxy

      ```
      public void setMaxy(int maxy)
      ```


    - #### getMinx

      ```
      public int getMinx()
      ```


    - #### setMinx

      ```
      public void setMinx(int minx)
      ```


    - #### getMiny

      ```
      public int getMiny()
      ```


    - #### setMiny

      ```
      public void setMiny(int miny)
      ```


    - #### getContour\_type

      ```
      public Particle.CONTOUR_TYPE getContour_type()
      ```


    - #### setContour\_type

      ```
      public void setContour_type(Particle.CONTOUR_TYPE contour_type)
      ```


    - #### getCurves

      ```
      public java.util.ArrayList<java.util.ArrayList<ConcavePoint>> getCurves()
      ```


    - #### toPolygon

      ```
      public java.awt.Polygon toPolygon()
      ```

      Convert the particle contour to a polygon.

      Returns:
      :   a `Polygon`


    - #### toString

      ```
      public java.lang.String toString()
      ```

      In general this functions produces an XML entry of the particle.

      **Overrides:**
      :   `toString` in class `java.lang.Object`


    - #### getHeight

      ```
      public double getHeight()
      ```


    - #### setHeight

      ```
      public void setHeight(double height)
      ```


    - #### getWidth

      ```
      public double getWidth()
      ```


    - #### setWidth

      ```
      public void setWidth(double width)
      ```


    - #### getOrientation

      ```
      public double getOrientation()
      ```


    - #### setOrientation

      ```
      public void setOrientation(double orientation)
      ```


    - #### getArea

      ```
      public double getArea()
      ```


    - #### setArea

      ```
      public void setArea(double area)
      ```


    - #### getPrimeter

      ```
      public double getPrimeter()
      ```


    - #### setPrimeter

      ```
      public void setPrimeter(double primeter)
      ```


    - #### getMajorAxisAnchorPoint1

      ```
      public java.awt.Point getMajorAxisAnchorPoint1()
      ```


    - #### setMajorAxisAnchorPoint1

      ```
      public void setMajorAxisAnchorPoint1(java.awt.Point MajorAxisAnchorPoint1)
      ```


    - #### getMajorAxisAnchorPoint2

      ```
      public java.awt.Point getMajorAxisAnchorPoint2()
      ```


    - #### setMajorAxisAnchorPoint2

      ```
      public void setMajorAxisAnchorPoint2(java.awt.Point MajorAxisAnchorPoint2)
      ```


    - #### getMinorAxisAnchorPoint1

      ```
      public java.awt.Point getMinorAxisAnchorPoint1()
      ```


    - #### setMinorAxisAnchorPoint1

      ```
      public void setMinorAxisAnchorPoint1(java.awt.Point MinorAxisAnchorPoint1)
      ```


    - #### getMinorAxisAnchorPoint2

      ```
      public java.awt.Point getMinorAxisAnchorPoint2()
      ```


    - #### setMinorAxisAnchorPoint2

      ```
      public void setMinorAxisAnchorPoint2(java.awt.Point MinorAxisAnchorPoint2)
      ```


    - #### removeDuplicates

      ```
      public void removeDuplicates()
      ```

      Remove duplicates points if found.


- Package
- Class
- Tree
- Deprecated
- Index
- Help

*org.biolag.particleprocessor 1.0*

- Prev Class
- Next Class

- Frames
- No Frames

- All Classes

- Summary:
- Nested |
- Field |
- Constr |
- Method

- Detail:
- Field |
- Constr |
- Method
